# Bilateral Myeloid Sarcoma of the Breast: A Case Report With Radiological and Pathological Correlation

**DOI:** 10.7759/cureus.24731

**Published:** 2022-05-04

**Authors:** Dana Amiraian, Michelle McDonough, Xochiquetzal Geiger

**Affiliations:** 1 Radiology, Mayo Clinic, Jacksonville, USA; 2 Laboratory Medicine and Pathology, Mayo Clinic, Jacksonville, USA

**Keywords:** breast cancer, leukemia, breast masses, myeloblastoma, extramedullary, sarcoma, myeloid

## Abstract

Myeloid sarcoma is a solid extramedullary mass of immature myeloid cells, often in patients with myeloid leukemia. Myeloid sarcoma of the breast is extremely uncommon, and bilateral involvement is even rarer. Myeloid sarcoma of the breast can mimic primary breast cancer, lymphoma, and other neoplasms. Differentiation between myeloid sarcoma and primary breast malignancy is imperative, as management and treatment are drastically different. We present a case of myeloid sarcoma of both breasts in a 63-year-old female with relapsed acute myelogenous leukemia (AML), a personal history of ovarian cancer, and a family history of both leukemia and breast cancer. This report highlights the need for high clinical, radiological, and pathological suspicion to diagnose myeloid sarcoma of the breast.

## Introduction

Myeloid sarcoma of the breast is an extremely rare entity that can easily be mistaken for other pathology. Myeloid sarcoma is a solid extramedullary mass of immature myeloid cells [1-4]. Other terms for myeloid sarcoma include chloroma, granulocytic sarcoma, monocytic sarcoma, myeloblastoma, and extramedullary myeloid cell tumor [1,5,6].

Myeloid sarcoma often occurs in patients with a known diagnosis of myeloid leukemia, although it can also be detected concomitantly or as the initial presentation of leukemia [1,2,5]. Myeloid sarcoma can present in various anatomical locations, most commonly in bone, lymph nodes, and skin [1,5,7,8]. However, myeloid sarcoma of the breast is uncommon and is usually unilateral [4].

We present a rare case of bilateral breast myeloid sarcoma. This case report adds to the limited literature on bilateral myeloid sarcoma of the breast and emphasizes the need for high clinical, radiological, and pathological suspicion to make this diagnosis.

This article was previously presented as a poster at the 2017 Florida Radiological Society Annual Meeting on July 23, 2017.

## Case presentation

A 63-year-old female with acute myelogenous leukemia (AML) and a history of treated ovarian cancer presented to our multidisciplinary breast clinic with a two-week history of rapidly enlarging palpable breast masses. She denied any other current complaints.

She was diagnosed with AML two years prior to presentation, initially achieving remission with chemotherapy. She subsequently relapsed one year later. Despite chemotherapy, she had residual leukemia on bone marrow biopsy and was currently undergoing evaluation for allogeneic bone marrow transplant.

She had also been diagnosed with ovarian cancer seven years prior to her current presentation and five years prior to her AML diagnosis. She had undergone debulking surgeries and chemotherapy for ovarian cancer and appeared to be in remission, without requiring treatment for over five years.

Family history was significant for breast cancer in the patient’s mother and maternal cousin and for leukemia in another maternal cousin. The patient’s estimated lifetime risk of breast cancer was elevated at 25% using the Tyrer-Cuzick model.

**Figure 1:**
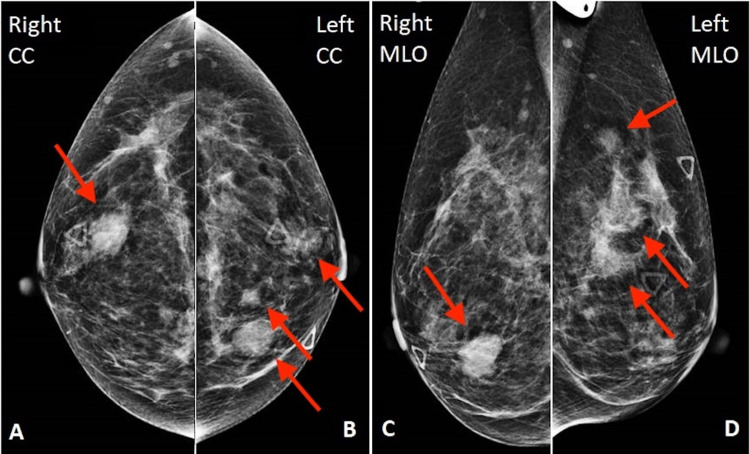
Bilateral mammography There is a single high-density, oval, irregular mass in the lower outer right breast (arrow in A and C) and multiple high-density, obscured, irregular masses in the upper inner left breast (arrows in B and D).

The patient underwent bilateral diagnostic mammography (Figure 1), which demonstrated multiple high-density, obscured, irregular masses in the upper inner quadrant of the left breast and a single high-density, oval, irregular mass in the lower outer quadrant of the right breast. Some of the left breast masses and the single right breast mass corresponded to sites of palpable concern.

**Figure 2:**
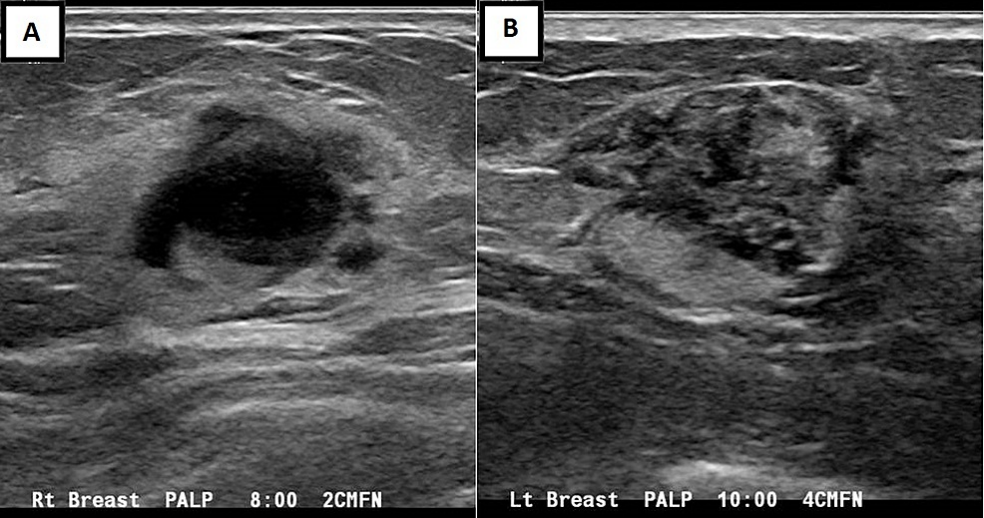
Bilateral breast ultrasonography Similar masses are seen in the right (A) and left (B) breasts with a complex hypoechoic central portion and a hyperechoic peripheral halo.

Subsequent bilateral breast ultrasound (Figure 2) demonstrated these masses to be similar in appearance with a complex hypoechoic central portion and a hyperechoic peripheral halo.

Ultrasound-guided core needle biopsy of the right breast mass and the largest left breast mass was performed and revealed myeloid sarcoma (recurrent myeloid leukemia) at both biopsy sites.

**Figure 3:**
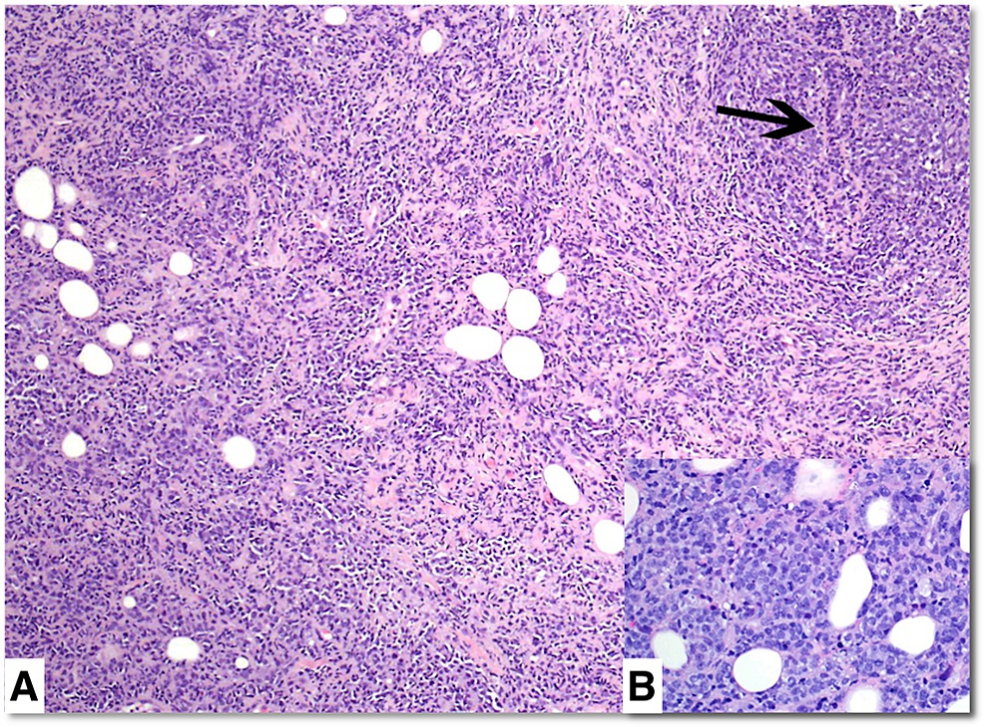
Hematoxylin-eosin stain of the core biopsy specimen Hematoxylin-eosin stain with original ×100 magnification (A) and right insert with original ×400 magnification (B). There is a dense neoplastic mononuclear infiltrate with small foci of residual benign ducts (arrow). Tumor cells have round to oval nuclei, dispersed chromatin, and occasional small nucleoli (B).

Core biopsies of both breast masses showed a dense diffuse mononuclear neoplastic infiltrate surrounding residual breast ducts and lobules with focal tumor infiltration of duct epithelium (Figure 3A). The tumor was composed of medium to large immature myeloid cells and myeloblasts with round to oval nuclei, dispersed chromatin, and distinct small nucleoli (Figure 3B). Frequent mitotic figures were present, but there was no necrosis.

**Figure 4:**
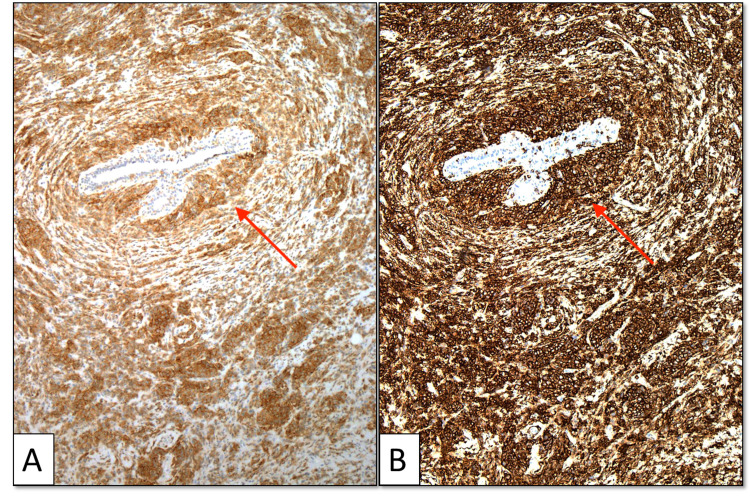
CD33 and CD43 immunohistochemistry of the core biopsy specimen CD33 and CD43 immunohistochemistry with original ×100 magnification. There is diffuse positive cytoplasmic staining of myeloid cells for CD33 (A) and CD43 (B). A tumor surrounds and focally infiltrates a benign breast duct (arrows).

**Figure 5:**
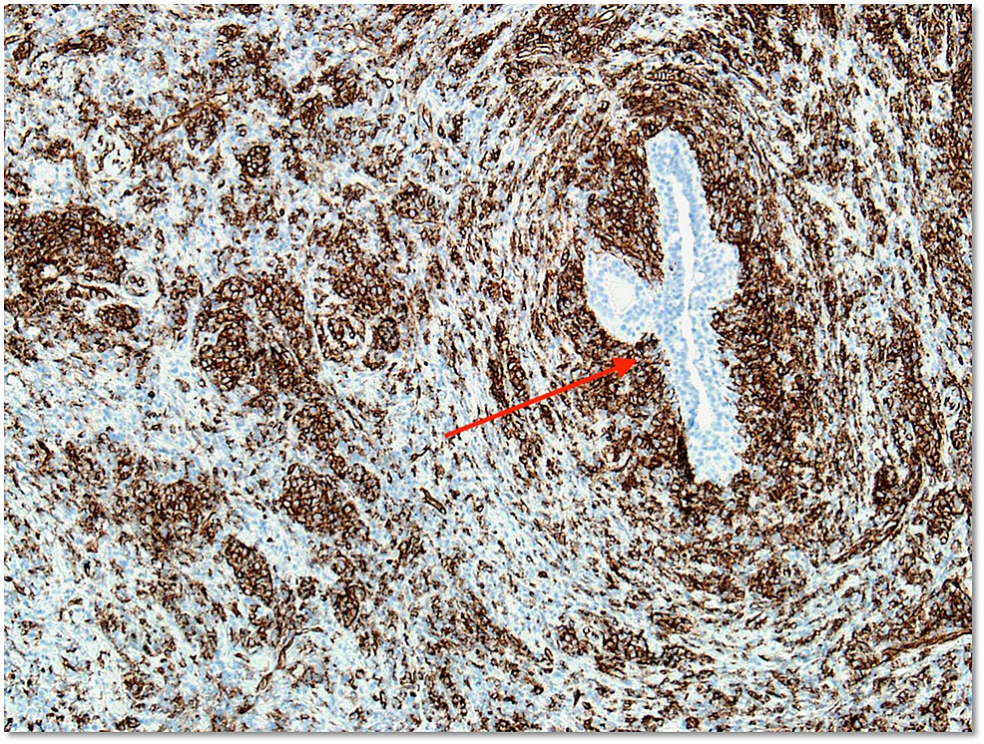
CD34 immunohistochemistry of the core biopsy specimen CD34 immunohistochemistry with original ×100 magnification. Many individual blasts and small blast clusters are highlighted by CD34 staining. A CD34-negative benign breast duct is present (arrow).

Myeloperoxidase, lysozyme, and pan-cytokeratin stains were negative. However, immunohistochemistry showed diffuse CD33 and CD43 tumor cell staining with the absence of CD3 staining, confirming myeloid lineage (Figure 4). Many myeloblast cell clusters were highlighted by CD34 (Figure 5). Myeloid sarcoma fluorescence in situ hybridization (FISH) testing confirmed monosomy 7 in 88% of nuclei, consistent with the patient’s previously identified AML clone.

Given the biopsy-proven myeloid sarcoma of both breasts, plans were made for salvage chemotherapy and future consideration of allogeneic bone marrow transplant. Unfortunately, the patient expired.

## Discussion

Myeloid sarcoma of the breast is extremely rare, and involvement of both breasts is even less common [4]. The vast majority of cases of myeloid sarcoma of the breast are in females, although cases in males with relapsed myeloid leukemia have been reported [9-11]. The age at diagnosis ranges from 16 to 73 years [4,6].

Myeloid sarcoma most commonly occurs in patients with a known diagnosis of myeloid leukemia. It can also occur concurrently with AML or as the initial presentation of relapse in patients with previously treated AML, including patients with prior allogeneic bone marrow transplantation [1]. Myeloid sarcoma can rarely be an isolated finding preceding blood or bone marrow evidence of myeloid leukemia [5,6].

Unfortunately, there are no classic imaging findings of myeloid sarcoma of the breast. Given its rarity and relatively nonspecific imaging characteristics, myeloid sarcoma of the breast can be misdiagnosed as primary breast malignancy, lymphoma, and other neoplasms or inflammatory processes [2,5,6]. The current case adds to the limited prior literature describing myeloid sarcoma of the breast as irregular and hypoechoic masses on imaging [2,4,6,7]. Since imaging is often similar in myeloid sarcoma as in other entities such as primary breast malignancy, suspicion for myeloid sarcoma will likely be based on patient history.

Pathologically, myeloid sarcoma typically has either a diffuse or single-cell infiltrating growth pattern. It is further classified based on cell maturation into mature, immature, or blastic subtypes [1]. The single-cell infiltration pattern of myeloid sarcoma can mimic the pathological appearance of invasive lobular breast carcinoma [2,6]. However, ductal and lobular breast structures are expected to be maintained in myeloid sarcoma [6]. Necrosis can help differentiate myeloid sarcoma from large cell lymphoma. Discrete regions of necrosis are common in large cell lymphoma, whereas necrosis is not a typical feature in myeloid sarcoma [6].

Immunohistochemistry is essential in diagnosing myeloid sarcoma. As in the present case, positive staining is often seen with CD43, CD34, and CD33 [1,6]. Myeloperoxidase and lysozyme are also usually positive [1,6], although they were negative in this case. CD3 staining should be negative [1], as in this case. Cytokeratin staining indicating epithelial cell lineage is also positive in ductal and lobular breast carcinoma but negative in myeloid sarcoma. Estrogen and progesterone receptor staining also would not be expected in myeloid sarcoma but can be seen in invasive breast carcinoma [2]. Chromosomal anomaly with monosomy 7 is rare but has been previously reported [1]. Given the vital role of immunohistochemistry and pathological testing in diagnosing myeloid sarcoma, it is imperative that the appropriate clinical information and suspicion for myeloid sarcoma be relayed from the radiologist to the pathologist when biopsy samples are submitted. This alerts the pathologist to collect tissue appropriately and perform additional testing to render this rare diagnosis.

The treatment of myeloid sarcoma of the breast is drastically different from that of primary breast cancer. Whereas surgery is the mainstay of breast cancer treatment, myeloid sarcoma is typically treated with chemotherapy, as well as stem cell transplant in some cases [1]. Surgery and radiation alone are usually inadequate [12]. Myeloid sarcoma of the breast usually has a poor prognosis, although there are cases of long disease-free survival among these patients [12]. This underscores the importance of early accurate diagnosis so that prompt and intensive treatment can be undertaken with the hope of long-term survival and cure, and unnecessary surgery or inappropriate neoadjuvant chemotherapy is avoided.

## Conclusions

Myeloid sarcoma of the breast is an extremely rare entity, and bilateral breast involvement is even rarer. This entity can therefore be misdiagnosed as other disease processes, including primary breast malignancy. However, myeloid sarcoma should be considered clinically, radiologically, and pathologically in patients with breast masses, particularly when there is a history of myeloid leukemia. A timely and accurate diagnosis of myeloid sarcoma of the breast is necessary for appropriate management, as treatment differs considerably from that of primary breast malignancy.
